# Developing an award program for children's settings to support healthy eating and physical activity and reduce the risk of overweight and obesity

**DOI:** 10.1186/1471-2458-9-345

**Published:** 2009-09-18

**Authors:** Suzy Honisett, Suzi Woolcock, Creina Porter, Ian Hughes

**Affiliations:** 1 *Kids - 'Go for your life'*, Diabetes Australia - Vic and Cancer Council Victoria, Melbourne, Australia; 2 Dynamic Outcomes Pty Ltd, St Andrews, Australia

## Abstract

**Background:**

This paper aimed to identify the best way to engage, motivate and support early childhood services (ECS) and primary schools (PS) to create policy and practise changes to promote healthy eating and physical activity. This information would be used to develop a suitable program to implement within these children's settings to reduce the risk of childhood overweight and obesity.

**Methods:**

The Medical Research Council's (UK) framework for the design and evaluation of complex interventions was used to guide the development of the healthy eating and physical activity program suitable for ECS and PS. Within this framework a range of evaluation methods, including stakeholder planning, in-depth interviews with ECS and PS staff and acceptability and feasibility trials in one local government area, were used to ascertain the best way to engage and support positive changes in these children's settings.

**Results:**

Both ECS and PS identified that they had a role to play to improve children's healthy eating and physical activity. ECS identified their role in promoting healthy eating and physical activity as important for children's health, and instilling healthy habits for life. PS felt that these were health issues, rather than educational issues; however, schools saw the link between healthy eating and physical activity and student learning outcomes. These settings identified that a program that provides a simple guide that recognises good practise in these settings, such as an award scheme using a health promoting schools approach, as a feasible and acceptable way for them to support children's healthy eating and physical activity.

**Conclusion:**

Through the process of design and evaluation a program - *Kids - 'Go for your life'*, was developed to promote and support children's healthy eating and physical activity and reduce the risk of childhood overweight and obesity. *Kids - 'Go for your life' *used an award program, based on a health promoting schools approach, which was demonstrated to be a suitable model to engage ECS and PS and was acceptable and feasible to create policy and practise changes to support healthy eating and physical activity for children.

## Background

Childhood overweight and obesity are significant public health issues. Recent prevalence data show 17% of Australian children (2-16 years) are overweight and 6% are obese [1]. These prevalence figures are predicted to increase, such that 34% of boys and 37% of girls 5-9 years of age will be overweight and/or obese by 2025 [2]. The current Victorian statewide prevalence of childhood overweight and obesity appear to be in keeping with national trends. However, current primary school aged figures vary across the state, with prevalence measured at 27% in one rural Victorian area [3] and higher (30%) in an inner urban, culturally diverse population [4].

Childhood obesity is an important predictor of adult obesity [5], leading to significant long-term health consequences. Rising levels of overweight and obesity produce an enormous burden through decreased life expectancy and reduced quality of life as a result of cardiovascular disease, type 2 diabetes, some types of cancer, sleep apnoea, osteoarthritis, psychological disorders and social problems [6].

To prevent obesity in the general population it is important to focus on children and their maintenance of healthy weight as an effective public health approach. Children's settings, such as primary schools, kindergartens, child care and family day care services, are key places that can enhance public health gains. These settings provide an opportunity to: reach children from all cultural and socio-economic backgrounds; expose children to nutritional and physical activity opportunities; involve parents in nutrition and physical activity education; and involve stakeholders from the broader community in the work of these settings, such as local dietitians to run parent healthy eating information sessions [7].

Systematic reviews of heterogenous school based interventions implementing strategies to improve healthy eating and/or physical activity present findings on their effectiveness that remain largely inconclusive, although they suggest a combination of nutrition and physical activity interventions may help prevent overweight [8] and assist in weight reduction [9]. Evidence from multi-facetted interventions within schools using a health promoting schools approach to increase healthy eating and physical activity behaviours in children show this is an effective approach to improve health knowledge and most likely to improve health related behaviour, such as dietary intake [10]. A health promoting schools approach, endorsed internationally by WHO, includes embedding healthy eating and physical activity within school policies, the schools' physical environment, curriculum and community links to ensure effectiveness and sustainability. The health promoting schools approach can be adapted and may be appropriate for early childhood services, such as kindergartens, child care and family day care.

Built within the health promoting schools approach, healthy award schemes have emerged. Award schemes provide a structured framework, health-related targets and provide external support. These schemes offer recognition for achieving key elements of a health promoting schools approach and can be used to motivate healthy changes and as marketing tool to position the school as a desirable choice for families. The award scheme has become popular among European countries to monitor systems and recognise achievement [11] and is present in some Australian schools and early childhood services. The evaluation of award schemes, so far have demonstrated award-related changes in terms of children's health behaviours and the schools healthy eating and physical activity policies and practises [12].

Within Victoria, Australia, primary school attendance is compulsory for children from five years of age. There is a range of education options available, including government, catholic and independent schools. Access to schools is not restricted by residential geography or zoning. Prior to school attendance, kindergartens provide a one-year education program for children aged four years and child care is available for children aged up to six years.

These children's settings are primarily focused on learning and development outcomes, as directed by government policy [13]. Therefore, the challenge is to develop a program that engages and motivates a majority of children's settings to effectively promote and support health behaviours that will reduce the risk of overweight and obesity in children.

The Victorian *Kids - 'Go for your life' *program aims to improve healthy eating and physical activity levels of children and in doing so reduce the risk of overweight and obesity. To achieve this aim *Kids - 'Go for your life' *works with primary schools and early childhood services, such as child care, family day care and kindergartens, to support healthy eating and physical activity through an award program, using a health promoting schools approach. The award program provides a comprehensive, yet simple, guide for early childhood services and schools to create healthy environments that support children to be active and eat well. Schools and services join the program as members and receive a range of resources, training and support as they work through a number of criteria to improve their policies and practices and reach award status. Once awarded schools and services receive a sign for their front gate to show to their community their commitment to children's healthy eating and physical activity.

The paper aims to describe the design of the *Kids - 'Go for your life' *award program operating in early childhood services and primary schools within Victoria and the methods that lead to its development.

## Methods and results

To develop an appropriate program for early childhood services and primary schools three phases of evaluation were undertaken. These phases were based on those of the Medical Research Council (UK)[14] framework for development and evaluation of complex interventions to improve health. These involved; a theoretical phase, identifying the most appropriate model; modelling phase, determining what components would make this model successful; and trialling phase, testing the feasibility of this model within settings. The formative evaluation methods and outcomes are described below for each of these phases.

These evaluation methods were approved through the Department of Human Services Victoria, Human Research Ethics Committee (54/07) and the Department of Education and Early Childhood Development Victoria, Research in Schools Committee.

### Theoretical phase

The initial phase of development aimed to answer the question - 'what is the most appropriate model for a physical activity and health-eating program for primary schools and early childhood services in Victoria?' A number of methods were employed to answer this question.

Eight key stakeholders, who had a vested interest and expertise in healthy eating, physical activity and childhood obesity, were involved in a strategic planning session. These stakeholders represented non-government health organisations, research institutes, a tri-partisan statutory health promotion organisation and a health consultant. The stakeholders identified key health outcomes that they envisioned the program should achieve in 10 years and listed gaps and opportunities in achieving these outcomes. From this process strategic themes were drawn from the discussions and the model of an award program for early childhood services and primary schools was proposed as an appropriate and achievable model to engage settings to make changes in policy and practice that would support healthy eating and physical activity. The success of programs such as SunSmart schools [15] and Be Active Eat Well [16] in engaging schools, promoting healthy school policies and practices and changing children's behaviours guided and supported this decision.

A subsequent brief review of published award or multi-strategy health promotion programs operating within children's settings across Australia and internationally was undertaken, to identify program designs, theoretical approaches used and successful features. Where possible discussions with key staff members of these programs assisted understanding of the key features of these programs that contributed to their success. Common features were identified across these programs and included; sustainability; utilising a health promoting schools approach; targeting specific behaviours; providing recognition to settings for their achievements; streamlining settings' access to on-the-ground support and resources; consulting with settings; provision of support through local partnerships; integration and support of settings' existing structures and requirements to reduce added burden; and the ability for settings to progressively work toward change.

The award model would also be required to support existing state and national policies for nutrition and physical activity. Relevant polices and regulatory requirements were identified and included; mandated provision of physical education and sport, and curriculum frameworks within government schools; national guidelines for non-government schools on the provision of physical activity; state licensing standards and national accreditation standards for early childhood services relating to nutrition and physical activity; and national government guidelines on physical activity and healthy eating for children 0-12 years.

The outcome of this phase was that an award program, that provided recognition to settings for their achievements and was based on a health promoting schools approach, was developed and implemented to engage and drive change within children's settings. This program would focus on improving healthy eating and physical activity policies and practises, be aligned with relevant national and state policies and consider the key success elements of other existing programs.

### Modelling phase

The modelling phase aimed to define the detail of the award program and any supporting materials. The initial methodology for this phase included twenty-six in-depth interviews with settings staff, including school principals (10), assistant principals (1), teachers (4) and early childhood centre managers and coordinators from family day care (5), long day care (3) and kindergartens (3) to ascertain:

• Underlying **rationale**, and therefore the marketing strategy, that would motivate settings to be involved in the proposed award program.

• **Features **that would appeal to settings to motivate them to be involved and create healthy eating and physical activity changes.

• **Feasibility **of the proposed design of the award program.

• **Support **that settings would require when undertaking the award program.

Snowball sampling was used to identify relevant interviewees from metropolitan, rural, high and low socio-economic areas, catholic, independent and government schools. All interviews were undertaken either in person or by telephone and taped. Interviews were then reviewed by three people to ascertain common themes emerging from interviewee responses under each of these question areas (see Table 1 for interview questions). The interviewee responses to the feasibility of the award program were further investigated through the trialling phase of evaluation.

**Table 1:** In-depth interview questions for early childhood service and primary school staff

**Key areas of investigation**	**Questions**
Rationale for involvement in the program	What underlying rationale/marketing angle would motivate your school/service to engage in a program that promotes healthy eating and physical activity?

	Of any health initiatives undertaken at your school/service that involve healthy eating/physical activity, what was the motivation behind taking action?

	What do you think would be the benefits to a school/service in improving children's physical activity and healthy eating? Are there any negative aspects?

Features that would motivate settings' involvement	What types of features would appeal to you in terms of motivating your school/service to sign up to this program?

	Can you comment on this design in relation to:
	• Would this help your school/service plan and implement changes?
	• Is it a clear design?
	• Are there things to remove or add?

	What form of recognition would you like for being a part of this program?

Feasibility of the proposed design of the award program	What might be some of the barriers to being involved in the program? (E.g. time required, perceived workload, perceptions of school's council, parents and friends, food services, teachers, students)

	Planning - what would you need to do or be prepared to do to implement the program in your school/service?

Support required to implement the program	What is the best way to get information about the program out to schools/services?

	What do you think is the best way to implement and maintain these changes in your school/service?

	Do policies already exist on healthy eating and physical activity? How capable do you feel your school/service is in developing policies for healthy eating and physical activity? How could this program assist?

#### Rationale

Early childhood services identified that healthy eating and physical activity were important issues for children's health, and instilling healthy habits for life provided a strong rationale for their involvement in an award program.

Many schools felt that obesity was not prevalent in their own student community and tackling obesity was considered a health issue, rather than an educational issue, which was identified as their core business. However, schools saw the link between healthy eating and physical activity and student learning outcomes, therefore improving these behaviours to increase educational outcomes provided a rationale for involvement in the award program. It was also important for schools that the award program be linked to existing policies and government requirements and not seen as additional or extra, but core to what they were required to do.

#### Features

Recognition, provided through certificates and other means, was identified as an important element by a majority of settings; however, schools did not want an award program to create competition between schools. While most settings said a sign on the front gate would be beneficial one school stated:

*"To be blunt, we don't go through these processes to put a sign on the gate, we are far too busy for that"*.

Professional development for staff and supporting resources, such as information for parents and curriculum support were identified as critical. Local support, such as linking with a local dietitian or school nurse, was also identified by schools as important.

#### Feasibility

There was positive feedback from early childhood services and schools about the perceived feasibility of the draft award program, with most identifying it as a clear and simple structure that was "very doable".

#### Support

It was identified that service managers' and principals' support would be essential to initially drive the award program. Within a school environment it was recognised that although the principal would initiate involvement in the program a champion teacher would be required to drive and implement the actions of the award program.

Expert opinion was gathered through an early childhood and a primary school stakeholder working group to comment on the award program design, including the key success features outlined in the theoretical stage, and criteria for the award program. Stakeholders in each of these groups represented key statewide child health, development and education organisations working in the early childhood and primary school sectors. The primary school working group also had a representatives of a government primary school.

Stakeholders supported the design and key elements of the award program; however, they reinforced essential considerations, such as consistency of terminology to reduce any confusion for settings, the provision of local support to assist settings, getting a balance between what is ideal and what is practical and ensuring equitable access and support for the program.

The working groups initially brainstormed possible strategies that could be implemented in early childhood services and primary schools. These strategies were grouped under each key healthy behaviour (increasing fruit, vegetable and water consumption, reducing consumption of high fat, salt and sugar foods and drinks, increasing participation in physical activity/active play, reducing sedentary behaviour, such as screen time, and increasing active transport) and under key health promoting schools approach elements, such as policy, information provided to parents and inclusion in curriculum and program planning. From the list of strategies grouped under each of these areas the two working groups chose one strategy, based on available evidence of effect, through their knowledge and in their experience, they felt was feasible and likely to produce the best behavioural outcomes. See additional file [Additional file 1] for the final award criteria.

It was important to engage stakeholders early in the process of defining the award program to ensure ownership and buy-in and subsequent promotion and support for the award program.

On completion of the design and selection of criteria, supporting resources were developed, based on the information from the in-depth interviews to ensure appropriate language and themes to engage and motivate settings. These resources were developed with extensive feedback from the early childhood and primary school working groups, who commented on each draft of the documents. These resources detail rationale for action, tips on how to make healthy changes, tips to support behaviour change with parents and other supporting resources and organisations.

The outcomes of the modelling phases confirmed the structure of the award program. This phase also assisted in choosing the best and most relevant criteria and developing supporting resources for settings.

### Trialling phase

The trialling phase aimed to explore the broader acceptability and feasibility of the award program for settings within a defined geographical area of Victoria and provide feedback and allow refinement of the model prior to statewide implementation.

The methods for this trial included sending letters to all primary schools (62) and early childhood services (84) residing in one outer metropolitan local government area of Victoria. This local government was chosen due to the high number of children's settings within the defined geographical area. Information on the socio-economic position and cultural diversity of children and families was only available at the local government level, rather than by suburb or school level, therefore this information was not available to compare schools or early childhood services involved in this feasibility trial and presents a limitation of this feasibility and acceptability data.

Each school and early childhood service within the trialling local government area were sent a letter promoting the *Kids - 'Go for your life' *award program and requesting that they join as members. Primary school principals within this local government area were also presented the award program and asked to join at a regular principals' network meeting. Subsequent reach of the program uptake was measured via the collation of membership forms received from schools and services. Membership forms also requested settings to self assess their current status against each award criteria. From these membership forms baseline policy and practise data were collected. Self-assessments against each award criteria was not validated, and therefore, poses a limitation to the validity of the baseline data.

Membership uptake was followed by semi-structured telephone interviews with key contacts from a sample of children's settings that had joined the award program as a member to determine reasons for involvement in the program, their comments on the award program design and criteria, and comment on supporting resources. Interviews were planned with 3 primary schools, 3 kindergartens and 3 child care centres. While the planned numbers of interviews were achieved with primary school and kindergarten contacts, only 2 child care centre contacts were interviewed. Of the available child care centre contacts there were only 2 that either consented to be interviewed or were available.

Semi-structured telephone interviews were also held with representatives from a sample of children's settings that had not joined the award program as a member to determine the reasons for not being involved and what would encourage them to join the award program. Interviews were conducted with representatives of 3 primary schools, 3 kindergartens and 3 child care centres.

Within four weeks of promoting the award program 26% of primary schools and 23% of early childhood services in the local government had joined the award program, demonstrating a reasonable level of early acceptability and feasibility for the award program (Table 2).

**Table 2:** Children's settings in trialing local government area that became members of the *Kids - 'Go for your life' *program

**Type of children's setting**	**Number of member settings**	**Percentage of each type of setting in local government area**
Primary schools	16	26%

Early childhood service	19	23%

Total	35	24%

For primary schools that had joined the program the criteria relating to restrictions on energy dense, nutrient poor canteen food (criteria 4) saw a relatively low percentage of schools self-assessing as having already achieved this (31%), or achieving it within 6 months (19%) (Table 3).

**Table 3:** Self-assessment against award program criteria for primary schools

**Criteria**	**Achieved** **%**	**Planned to be achieved within 6 mths** **%**	**Planned for the future** **%**	**Not planned at this stage** **%**	**NA** **%**	**No response** **%**	**Total** **%**
1. The school has a strategy in place to promote drinking water throughout the school day, especially during physical activity (eg water bottles). Only water is permitted in classrooms.	81.3	18.8	0.0	0.0	0.0	0.0	100.0

2. The school has a strategy in place to encourage fresh fruit and vegetable consumption every day in school breaks (eg fresh fruit/veg break or crunchy fruit and veg play lunch).	62.5	25.0	6.3	6.3	0.0	0.0	100.0

3. High sugar drinks (eg soft drinks) are excluded from the canteen/lunch order menus, other food services and vending machines (if applicable). Students and parents are requested not to bring these drinks to school.	50.0	18.8	25.0	6.3	0.0	0.0	100.0

4. The sale of chips, lollies, chocolate and fried foods are restricted from canteen/lunch order menus, other food services and vending machines. Students and parents are requested not to bring these foods to school.	31.3	18.8	43.8	6.3	0.0	0.0	100.0

5. Play equipment that encourages physical activity is made available to students at lunchtimes and breaks (eg balls, skipping ropes, bats).	81.3	6.3	12.5	0	0.0	0.0	100.0

6. Prep to Grade 6 students participate in 20-30 minutes of physical education per day (on average).	56.3	18.8	12.5	6.3	0.0	6.3	100.0

7. A whole school curriculum plan consistent with the Victorian Essential Learning Standards that promotes healthy eating and physical activity is in place.	37.5	37.5	18.8	6.3	0.0	0.0	100.0

8. The school promotes healthy, active and safe travel through a whole of school activity (eg Walking school bus, travel planning, walk and ride to school days, a walking challenge) at least one day per term.	31.3	31.3	25.0	12.5	0.0	0.0	100.0

9. Parents and caregivers receive regular updates on healthy eating and physical activity (eg newsletter inserts, parent information sessions) which include recommendations, ideas and strategies to support children's health and wellbeing.	43.8	25.0	18.8	12.5	0.0	0.0	100.0

Development of a whole school curriculum plan consistent with statewide standards (criteria 7) and the promotion of active travel (criteria 8) also saw relatively low percentages of schools self-assessing as having already achieved these (38% and 31% respectively). Within six months, providing the self-assessments are accurate, the majority of schools indicated they would have achieved these criteria (Table 3).

The majority of early childhood settings reported in their self-assessments either having already achieved, or planning to achieve within 6 months, most of the program criteria. Ensuring nutritious meals and snacks (criteria 1) had the lowest percentage of settings claiming to have achieved this already (61%), but the majority was planning to have achieved this within 6 months (Table 4).

**Table 4:** Self-assessment against award program criteria for early childhood services

**Criteria**	**Achieved** **%**	**Planned to be achieved within 6 mths** **%**	**Planned for the future** **%**	**Not planned at this stage** **%**	**N/A** **%**	**No response** **%**	**Total** **%**
1. Drinking water is available indoors and outdoors at all times and accessible to children (eg water bottles/water cooler/jugs).	94.7	5.3	0.0	0.0	0.0	0.0	100.0

2. Meals and snacks provided by the service and/or from home are nutritious and contribute to meeting the children's daily dietary and developmental requirements:							

A. Fresh fruit and vegetables are provided every day in the menu planning and encouraged in lunchboxes.	68.4	10.5	5.3	5.3	0.0	10.5	100.0

3. Positive meal environments are planned to be relaxed, social and enjoyable by:							

A. Children participating in serving, self feeding and sharing together	100.0	0.0	0.0	0.0	0.0	0.0	100.0

B. Encouraging children to try new foods regularly including different colours, textures, flavours and aromas	94.7	0.0	0.0	5.3	0.0	0.0	100.0

C. Providing the opportunity for staff/carers to sit with children when they are eating and drinking for role modelling, safety and socialisation	100.0	0.0	0.0	0.0	0.0	0.0	100.0

4. The following drinks and foods are not included in menu planning and are discouraged in lunchboxes. These include:							

A. Soft drinks, flavoured mineral waters, sweetened flavoured milks, cordials, 100% juice, fruit juice drinks and vitamin C syrups. Only plain milk and water are offered.	78.9	10.5	5.3	0.0	0.0	5.3	100.0

B. Pre-packed items such as chips, chocolates, lollies and muesli and fruit bars.	52.6	36.8	5.3	0.0	0.0	5.3	100.0

5. Food is not used as a reward, an incentive or for comforting children.	94.7	0.0	5.3	0.0	0.0	0.0	100.0

6. Program plans regularly incorporate a variety of indoor and outdoor physical activities such as dance, drama, moving to music and active games. These are planned to encourage all children and cater for a range of abilities.	94.7	5.3	0.0	0.0	0.0	0.0	100.0

#### Reasons for involvement in the program

Interviews with representatives from participating schools reported that they saw benefits for students joining the award program. All reported that their school had a commitment to encouraging healthy eating and physical activity for students and many had relevant initiatives happening at their school.

"When you work with kids, your interests lie with the kids, so it was a direct benefit to the students. Also we are experiencing a fair bit of social, cultural and economic disadvantage - for many kids, the model they have at home is not always a strong one as far as eating."

Interviewees from early childhood services reported they were more interested in improving the health of the children in their care than in recognition for their efforts through an award program, and this was their motivation for being involved.

#### Comments on the award program design and criteria

All interviewees felt that the award program and the material supplied were clear and well designed. All liked the idea of the award program, and in some cases as a form of recognition of what the school was doing:

*"I think it gives the parents something to look at and the parents are grateful that we're trying to support what they're doing at home. I find a lot of the parents are having difficulty trying to get children to eat correctly in the first place, and having the support at school makes it that bit easier for them"*.

One interviewee, the Principal of the school, provided an interesting perspective on the award program*, "Schools become very isolated, very insular, even though you might have a school no more than 3 or 4 blocks away, you're very isolated because schools have been forced to become very competitive. So you don't always get to know how you're going against what's happening in the broader scene. So it's useful for benchmarking..."*.

Two school interviewees felt that they would be able to meet all the criteria in the award program within 12 months. The other school felt that achieving the criteria wouldn't be difficult except for the canteen changes required.

Overall the award program and materials received were considered clear and easy to understand. All of the early childhood setting interviewees felt that the criteria in the award program were very achievable, and that they were already well on the way to meeting most:

*"I think I ticked most of them. I think there were only a couple that I didn't tick"*.

#### Feedback from settings not yet involved

##### Primary schools

All Principals interviewed were interested in the award program and could see that it fitted with existing initiatives and programs the schools were involved in:

*"A lot of what we are already doing fits in with the focus of Kids - 'Go for your life' as well. It would just be a matter of acknowledging that we are already doing a lot of things that fit in with the program"*.

Two of the Principals pointed to the difficulty of taking on something additional at this stage and felt that it was something they would take on later. One spoke about a major rebuilding project underway at present, including a new gymnasium, which was occupying his time and also that of the Physical Education Coordinator. The consistent theme was competing priorities at this point in time:

*"We have a number of competing priorities to implementing something new at this stage. It is certainly something that is worthwhile, but needs to be implemented in a coordinated way"*.

##### Early childhood settings

All interviewees expressed an interest in getting involved, but reported a variety of reasons for not joining as a member:

"We haven't discussed it as a staff, and so haven't decided yet". (Pre-school)

"We have put it to our committee of management and the staff are reviewing it to make sure we meet the criteria. It was talked about at the meeting last month, but we are scheduled to meet again in a couple of weeks. Our cook, who is here part-time, wants to get involved with it. So it is something we definitely want to do, it is obviously just practicalities of actually getting the paper work done". (Child care centre)

In one case the coordinator/teacher was leaving the pre-school shortly and felt that it would not be appropriate to commit her replacement to something like *Kids - 'Go for your life'*, but was planning to refer the information on. In another case the coordinator of one child care centre was shortly taking leave for an overseas holiday, but thought it would be something she would address when she returned.

### *Kids - 'Go for your life' *award program design

Victorian early childhood services and primary schools volunteer to become a member of the *Kids - 'Go for your life' *award program. To become a member, they are required to fill in a member form identifying what criteria they have already achieved, or are planning to achieve. The manager of the early childhood service or principal of the primary school signs the member form making a commitment to work toward reaching each award criteria (see Additional file 1 for the list of criteria). Early childhood services and schools are then sent comprehensive information packs detailing rationales and ideas to assist them to meet each criterion.

When settings achieve all criteria they complete and lodge an application form with supporting documentation (policies, menus, copies of newsletters sent home to families). If all criteria are met the setting will be awarded with a large metal outdoor sign to hang on their front fence to show that they are a *Kids - 'Go for your life' *service or school. Local media is offered to the setting to promote their achievements to their community.

The *Kids - 'Go for your life' *award program is promoted to Victorian early childhood services and primary schools through a range of public relations and communications activities, including, print community service announcements and articles in relevant journals and newsletters, local and statewide media, presentations at relevant forums and conferences and cross promotion with organisations and programs promoting similar or supporting messages.

Member and awarded settings are also provided with a range of additional supports. These supports include links to local health professionals who are available to support them to reach each criteria and become awarded, and in 10 local government areas *Kids - 'Go for your life' *have funded positions within local governments to support local settings to become members and work toward becoming awarded.

## Discussion

There are many examples of programs promoting healthy eating and physical activity within schools, using a health promoting schools approach [10], or award schemes [11] and, therefore, many of these interventions will be implementing similar strategies to the *Kids - 'Go for your life' *award program. However, to the best of our knowledge there is no published evidence of using a health promoting schools approach or award schemes supporting healthy eating and physical activity within early childhood services. One recently published prevention study investigated a comprehensive multi-strategy approach through kindergarten settings [17] using social learning theory. This study intervention included many elements of a health promoting schools approach; however, it did not include healthy eating and physical activity within kindergartens' policies, a key element of a health promoting schools approach. Therefore, the *Kids - 'Go for your life' *award program provides an innovative approach within an early childhood setting, using well-established components of the health promoting schools approach embedded within an award scheme.

The formative evaluations that inform the development of programs within schools or early childhood services are not often published, so the motivations and rationales for these settings' involvement are not well known or documented. While this was only a small-scale evaluation, it provided important information on: children's settings' motivations and rationales to become involved in healthy eating and physical activity programs; how the *Kids - 'Go for your life' *award program has been developed; and the acceptability and feasibility of this program by children's settings.

There has clearly been a level of success through the award program developed to encourage children's settings to join the program in the pilot local government area. Success with primary schools was greater than that with early childhood settings, therefore, the award program may need to be promoted and 'sold' more actively to these settings.

Concern for the health of children in their setting was the main reason for early childhood services and primary schools to become a member rather than the recognition through the award program. In this sense the program is seen as a way of bringing about change and obtaining support to do this. There was generally a positive reaction to the award program, criteria and materials received.

The feedback gathered through interviews with representatives of children's settings that had not responded to the invitation to join the program, indicates that there is a high degree of interest in the program. It is clear, however, that there are a number of competing priorities for settings and reasons for delaying involvement. As a result the need for ongoing promotion targeted individually to settings and through local media is critical, to ensure settings that are committed to other priorities at the moment are reminded of the program into the future.

It is significant that the majority of children's settings that joined as members had generally scored themselves quite well against the award program criteria in their self-assessments. This is particularly the case with the early childhood settings, which mostly saw themselves as either having already achieved, or planning to achieve within 6 months, most of the award program criteria. This may reflect an overestimation of settings current status and is a limitation of this evaluation. However, it is likely that the program has been successful in attracting those settings that are already committed and working actively to improving the health of children in their care, through improved nutrition and increased physical activity and active play. The challenge will be to also secure participation of those settings less willing, or feel less able, to join the program.

## Conclusion

Through the process of design and evaluation outlined in this paper *Kids - 'Go for your life' *was developed to promote and support children's healthy eating and physical activity and reduce the risk of childhood overweight and obesity. *Kids - 'Go for your life' *used an award program, based on a health promoting schools approach, which was demonstrated to be a suitable model to engage early childhood services and primary schools and was acceptable and feasible to create policy and practise changes to support healthy eating and physical activity for children.

The upcoming evaluation for the *Kids - 'Go for your life' *award program will now focus on the reach of the program across a range of demographic categories, including rural and metropolitan areas and areas of low socioeconomic positions. A major focus of the evaluation will also determine if and how the award program affects the policies and practices within children's settings and whether these changes impact on children's healthy eating and physical activity behaviours.

## Competing interests

The authors declare that they have no competing interests.

## Authors' contributions

SH, SW, CP and IH have made substantial contributions to conception and design, acquisition of data and analysis and interpretation of data. SH, SW, CP and IH have all been involved in drafting the manuscript, revising it critically for important intellectual content and have given final approval of the version to be published.

## Pre-publication history

The pre-publication history for this paper can be accessed here:



## Supplementary Material

Additional file 1 **Kids - 'Go for your life' award program criteria**. The table included lists all criteria for primary schools and early childhood services to become awarded. Click here for file
