# Supplementary material for: Developing an award program for children's settings to support healthy eating and physical activity and reduce the risk of overweight and obesity
Source: BMC Public Health. 2009 Sep 18;9:345. doi: 10.1186/1471-2458-9-345 (PMC2754461; doi:10.1186/1471-2458-9-345)
Supplement: Additional file 1 — Kids - 'Go for your life' award program criteria. The table included lists all criteria for primary schools and early childhood services to become awarded. [file 1471-2458-9-345-S1.DOC]

Additional file 1 - *Kids - 'Go for your life'* award program criteria

| **Award criteria** | **Kindergartens, child care and family day care** | **Primary schools** |
| --- | --- | --- |
| Increase water consumption | Drinking water is available indoors and outdoors at all times and is accessible to children (e.g. water bottles, water coolers, jugs). | The school has a strategy in place to encourage all students to drink water throughout the school day, especially during physical activity.  Only water is permitted for drinking in class time. |
| Increase fruit and vegetable consumption | Meals and snacks provided by the service and/or from home contribute to meeting children’s daily dietary and developmental requirements:   - Fresh fruit and vegetables are provided every day in the menu planning and - Encouraged in lunchboxes. | The school has a strategy in place to encourage fruit and vegetable consumption while at school (E.g. a fruit + veg break at 10am). |
|  | Positive meal environments are planned to be relaxed, social and enjoyable learning experiences by:   - Children participating in serving and self-feeding. - Encouraging children to try new foods regularly, including different colours, textures, flavours and aromas. - Providing the opportunity for staff /carers to sit with children when they are eating and drinking for role modeling, safety, learning and socialisation. |  |
| Reduce consumption of energy dense, nutrient poor foods and drinks | The following drinks and foods are not included in menu planning and are discouraged in lunchboxes:   - Soft drinks, flavoured mineral waters, sweetened flavoured milks, cordials, 100 percent juice, fruit juice drinks and vitamin C syrups. - Only water and plain milk are offered. - Pre-packaged items such as chips, chocolates, lollies, muesli and fruit bars. | Confectionery and high sugar drinks, such as soft drinks, energy drinks and flavoured mineral waters are excluded from the canteen and other school food services, including vending machines (if applicable). School community members are encouraged not to bring these items to school. |
|  | Food is not used as a reward, incentive or for comfort. | Chips (crisps) and fried foods are supplied no more than two times per term through the canteen or other school food services. School community members are encouraged not to bring these items to school. |
| Increase participation in physical activity | Daily structured and free active play is a significant component of the program plan. | The timetabling of physical activity, physical education and sport is consistent with appropriate education sector requirements and guidelines. |
| Reduce sedentary behaviour | Screen time (television/DVD/computers/electronic games) is not used or is limited in the service.  If screen time is used staff need to ensure it is planned, limited to a minimal part of the child’s day, is age appropriate and monitored, with staff sitting with children to discuss what is being viewed. | Play equipment that encourages physical activity is made available to students at lunchtime and other breaks. |
| Increase participation in active transport | Age appropriate traffic safety education, including pedestrian and car safety and playing safely, is provided as part of the program to children and their parents/carers in the service. | The school promotes walking or riding through a whole-of-school activity at least one day per term. |
| Utilise a Health Promoting schools approach | To ensure a sustainable whole of service approach:   - All *Kids – ‘Go for your life’* criteria are reflected in the service policies. - Families are informed of the policies. - Families are provided with regular information, ideas and strategies to promote healthy eating and active play. | The school has a whole school curriculum plan, which reflects the Victorian Essential Learning Standards, that encourages healthy eating and daily physical activity during and outside school hours.  Policy: the *Kids – ‘Go for your life’* Award criteria are included within policy and planning documents endorsed by the school council, or board, as appropriate.  Families are informed of healthy eating and physical activity policies and provided with information to assist them to meet policy requirements. |
